# Supplementary material for: Proteome-wide systems genetics identifies UFMylation as a regulator of skeletal muscle function
Source: eLife. 2022 Dec 6;11:e82951. doi: 10.7554/eLife.82951 (PMC9833826; doi:10.7554/eLife.82951)
Supplement: Figure 5—source data 1. — The top corner of each membrane is cut above lane 1. [file elife-82951-fig5-data1.zip › Figure 5A-Source data/Figure 5A-source data.pdf]

Figure 5A - Source Data

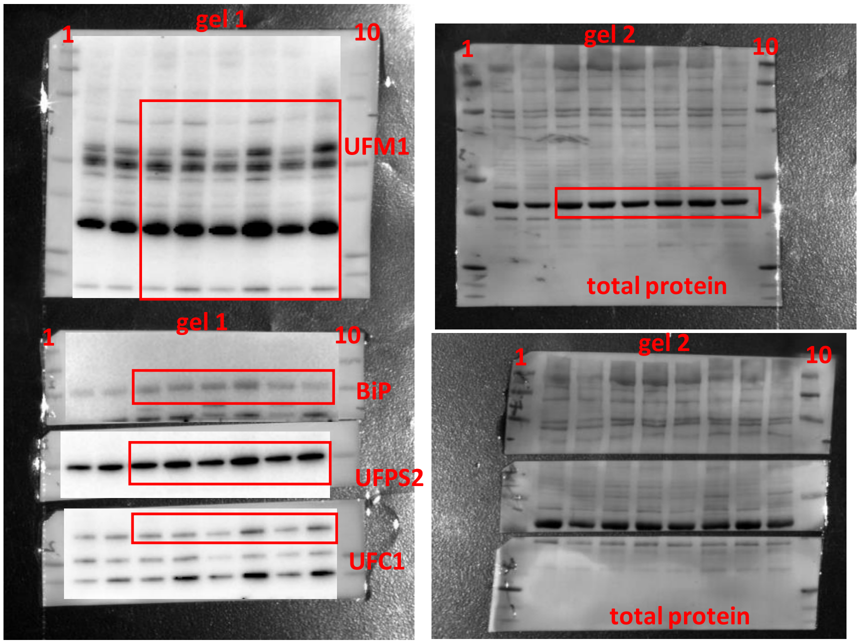

lane	Mouse ID #	Genotype
1	MW marker	
2	564	Non-Tg
3	566	SOD1G37R
4	565	Non-Tg
5	567	SOD1G37R
6	570	Non-Tg
7	568	SOD1G37R
8	571	Non-Tg
9	569	SOD1G37R
10	MW marker	

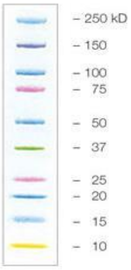

Gel 1

Whole gel

anti-UFM1 (Ab109305; Rb; 1:1000)

Gel 2

cut **under** 75, at 37

anti-BiP (CST3177; 1:1000; RB; 80kDa)
anti-UFSP2 (ab192597; Rabbit; 1:1,000; 53KDa)
anti-UFC1 (Ab189251; Rabbit; 1:10,000; 20kDa)

Mous ID#		UFC1	UFSP2	Conjufated-UFN	free-UFM1	BiP	Total	UFC1/Total	UFSP2/Total	Conjufated-UFN	free-UFM1/tota	BiP/total
564	Non-Tg	3348.104	8261.196	8631.125	4901.205	3430.945	17829.054	0.187789212	0.463355824	0.484104485	0.274899891	0.192435617
566	SOD1G37R	7422.397	10682.347	11695.64	6730.782	2951.167	13188.518	0.562792347	0.809973266	0.886804719	0.510351656	0.223767902
565	Non-Tg	5636.64	11019.418	10673.054	5870.933	5079.803	19563.518	0.288119959	0.563263622	0.545559035	0.300095975	0.259656929
567	SOD1G37R	13696.933	12472.761	13329.004	13057.631	4828.711	13550.225	1.010826979	0.920483682	0.983673998	0.963646803	0.356356518
570	Non-Tg	5516.912	8534.296	7083.69	4846.104	4438.497	15513.761	0.35561409	0.55011135	0.45660688	0.312374543	0.286100643
568	SOD1G37R	18340.317	14628.64	16667.054	15188.761	7052.418	14994.225	1.223158716	0.97561828	1.111564886	1.012974062	0.470342282
571	Non-Tg	5222.447	8056.397	6276.175	4814.761	3936.368	19681.225	0.265351725	0.409344286	0.318891482	0.244637262	0.20000625
569	SOD1G37R	15308.296	14240.037	16317.711	12875.66	3664.418	16350.104	0.936281262	0.870944735	0.998018789	0.787497132	0.224121999
