# Supplementary figures and images for: Proteome-wide systems genetics identifies UFMylation as a regulator of skeletal muscle function
Source: eLife. 2022 Dec 6;11:e82951. doi: 10.7554/eLife.82951 (PMC9833826; doi:10.7554/eLife.82951)

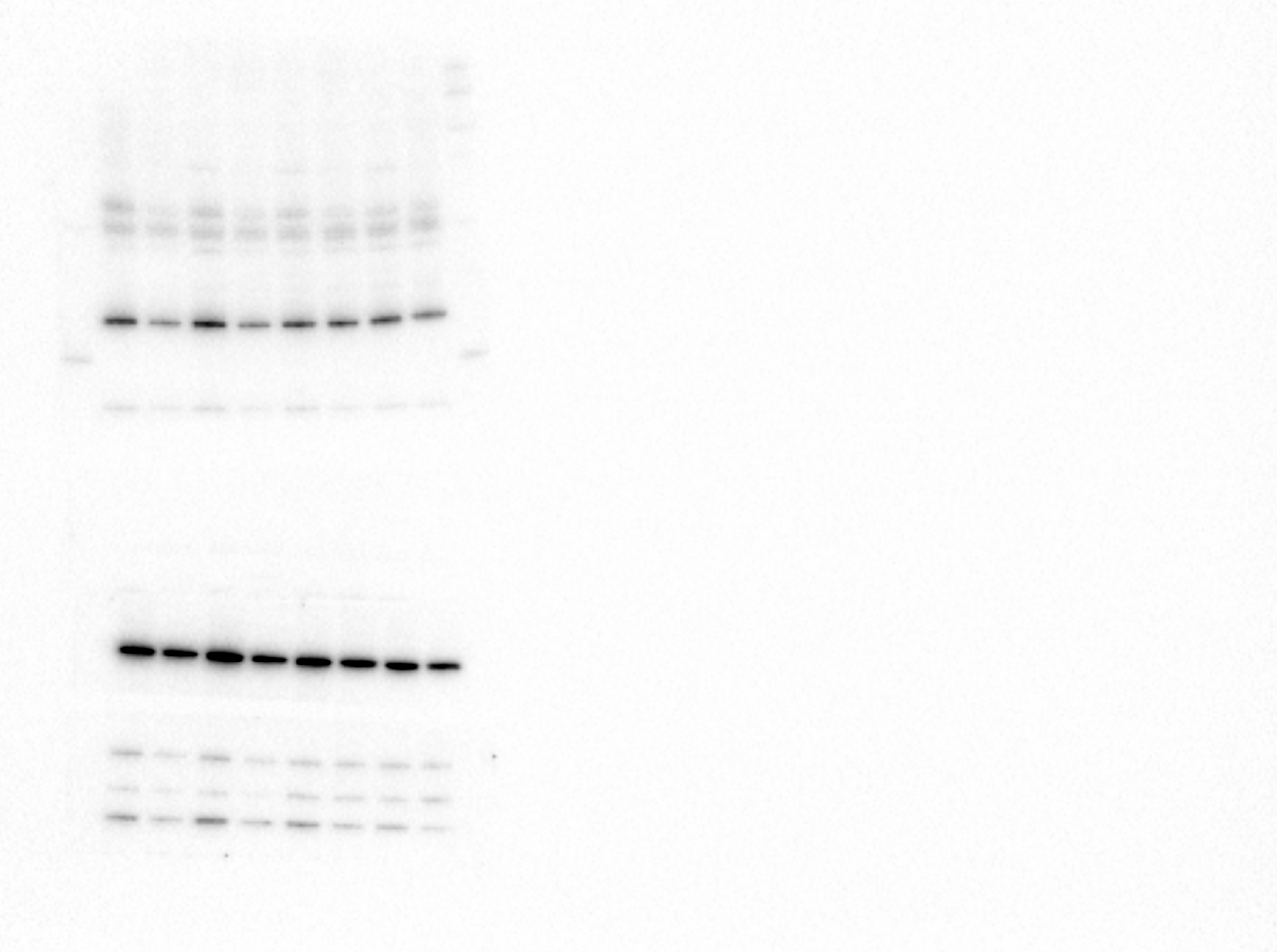

Supplement: Figure 5—source data 1. — The top corner of each membrane is cut above lane 1. [file elife-82951-fig5-data1.zip › Figure 5A-Source data/Ben Parker 2022-03-16 15hr 10min_Exposure_4.0sec.tif]

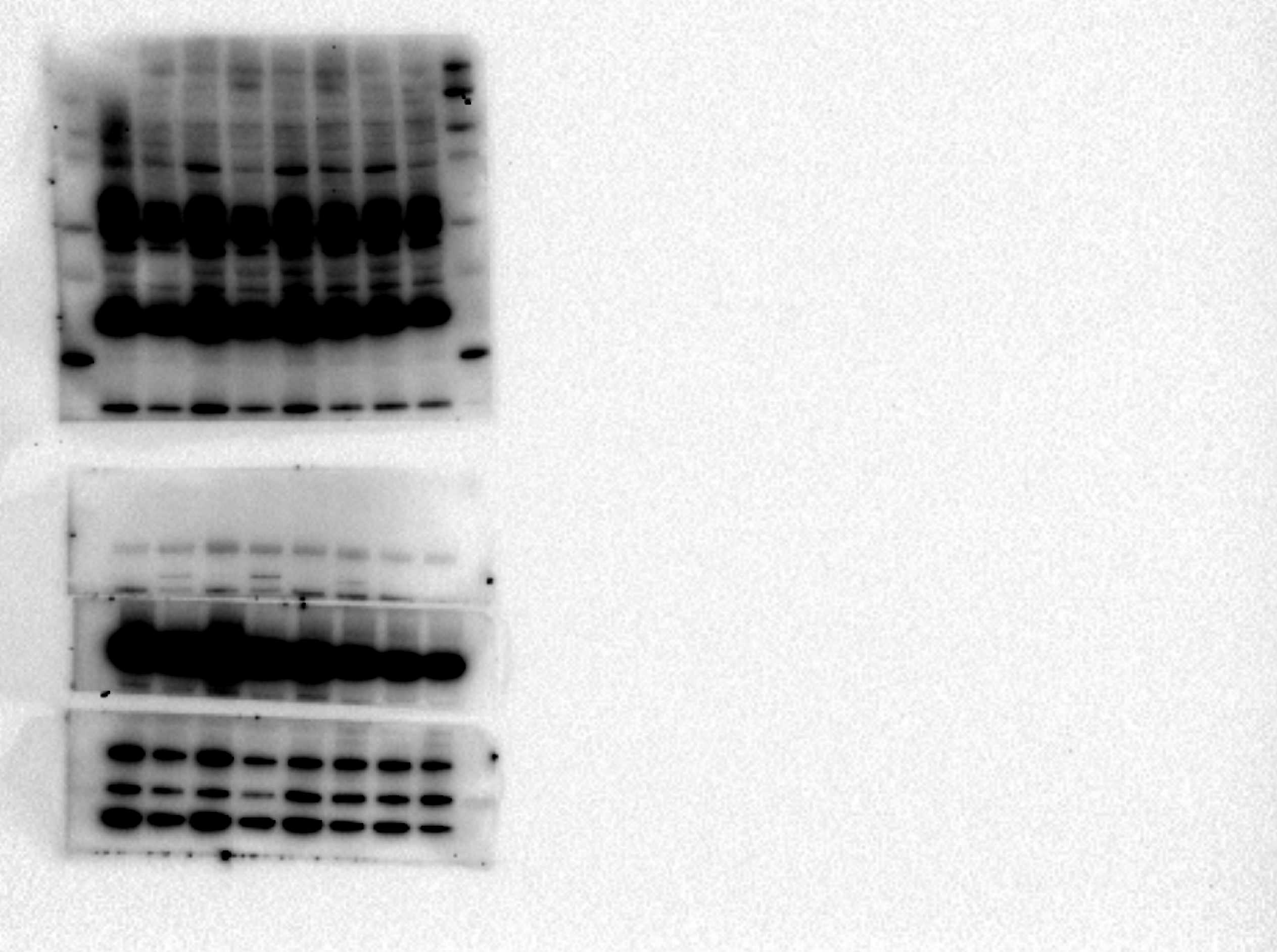

Supplement: Figure 5—source data 1. — The top corner of each membrane is cut above lane 1. [file elife-82951-fig5-data1.zip › Figure 5A-Source data/Ben Parker 2022-03-16 15hr 15min_Exposure_120.0sec.tif]

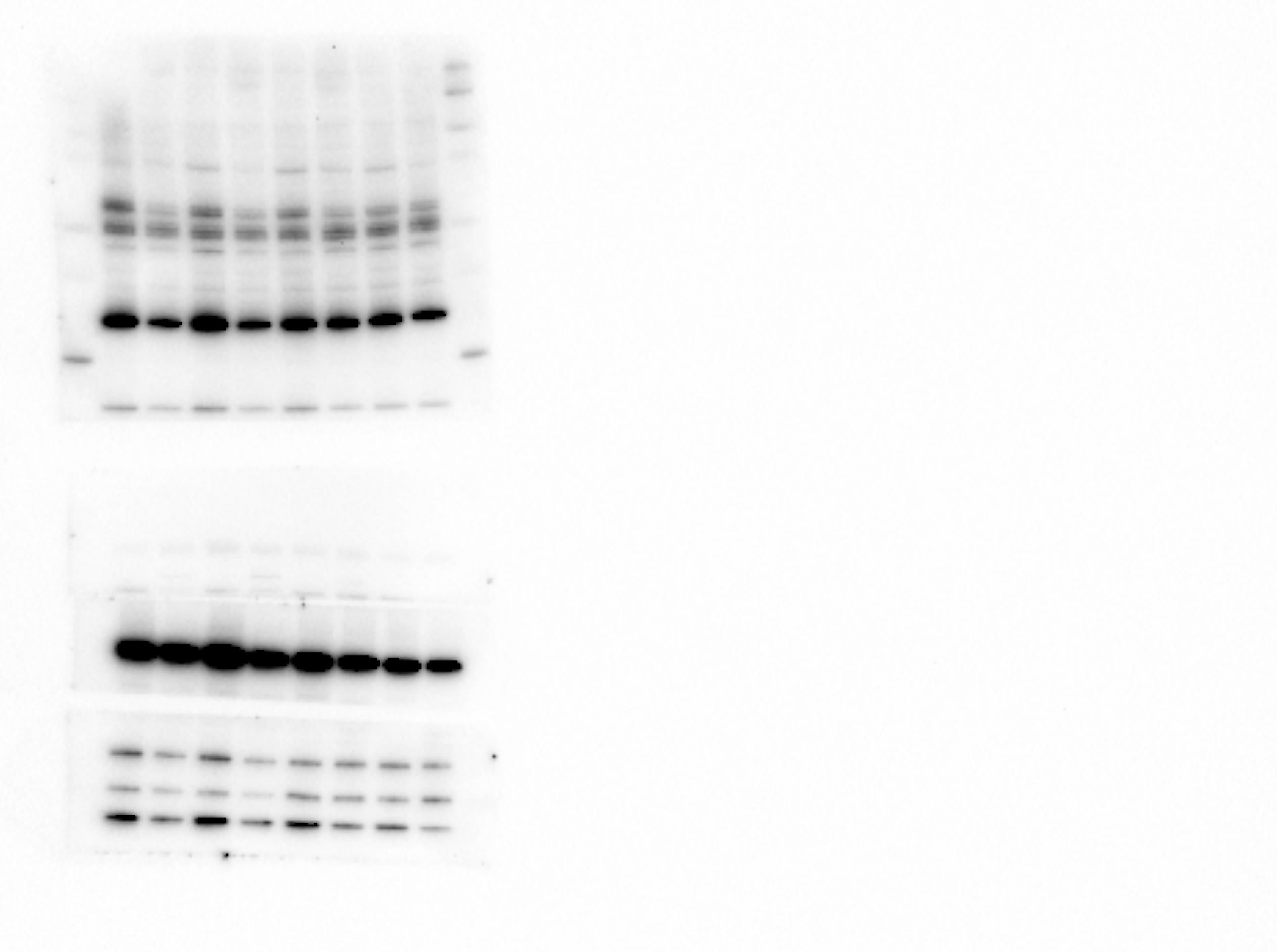

Supplement: Figure 5—source data 1. — The top corner of each membrane is cut above lane 1. [file elife-82951-fig5-data1.zip › Figure 5A-Source data/Ben Parker 2022-03-16 15hr 15min_Exposure_27.4sec.tif]

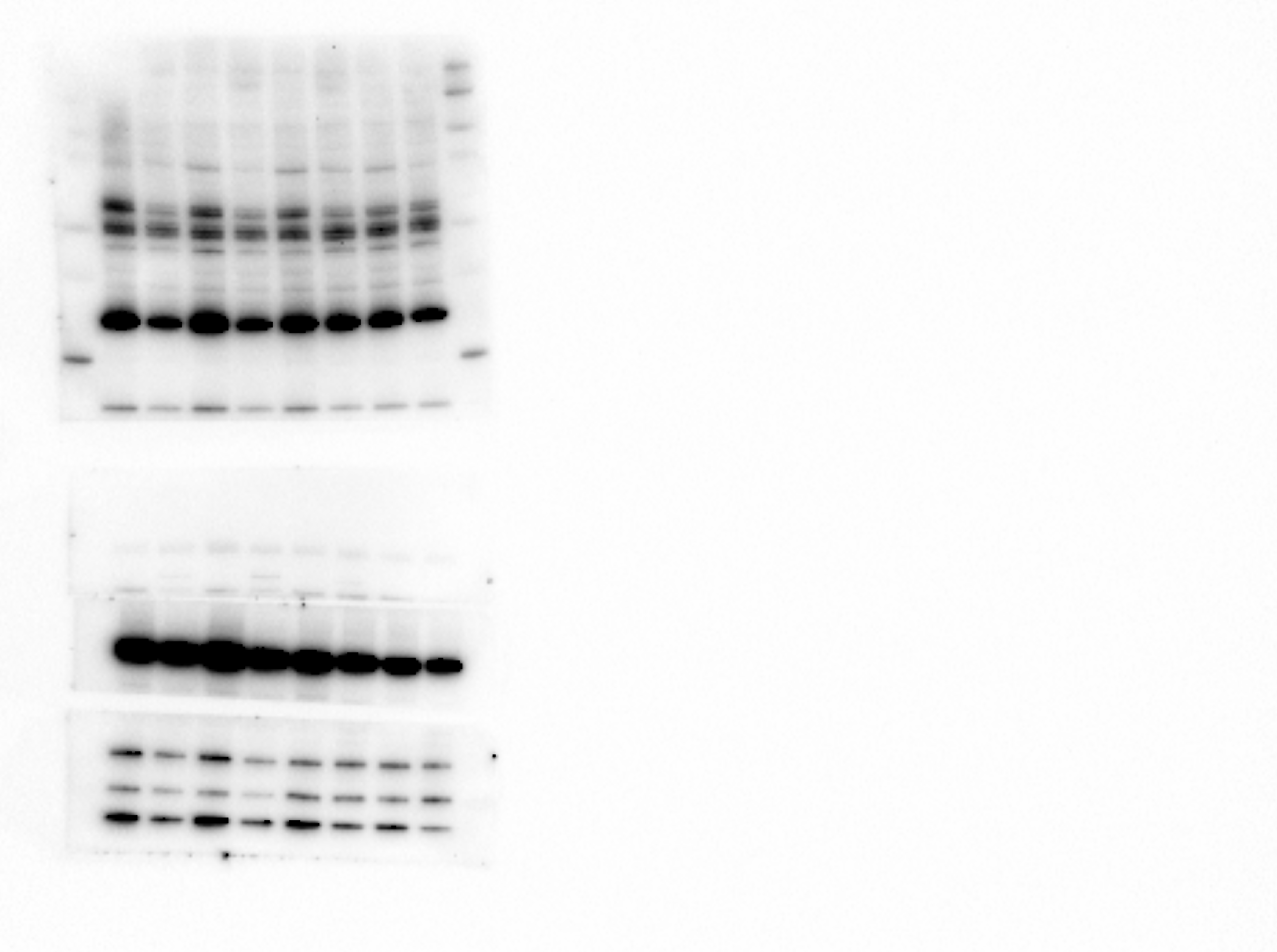

Supplement: Figure 5—source data 1. — The top corner of each membrane is cut above lane 1. [file elife-82951-fig5-data1.zip › Figure 5A-Source data/Ben Parker 2022-03-16 15hr 15min_Exposure_40.7sec.tif]

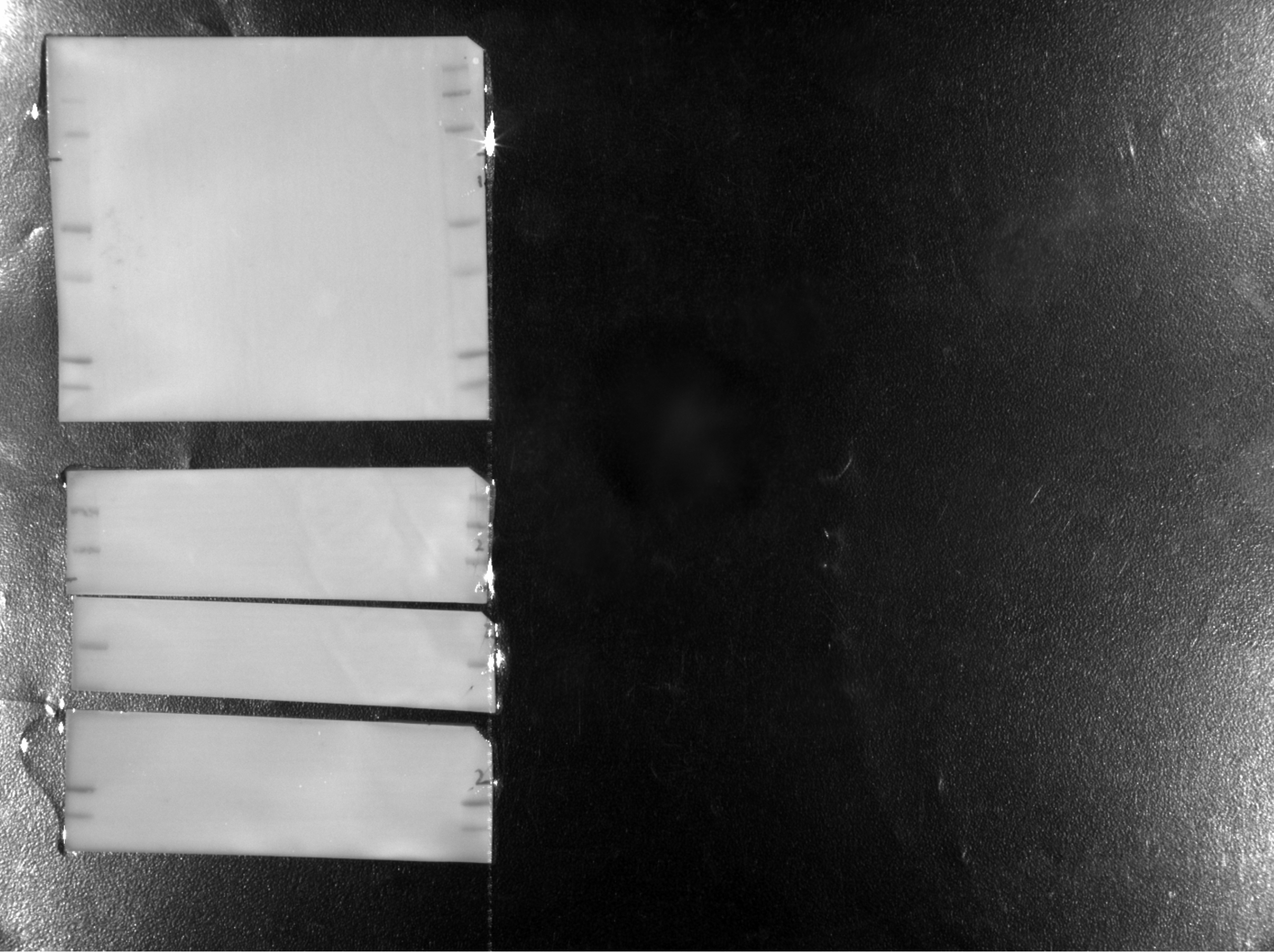

Supplement: Figure 5—source data 1. — The top corner of each membrane is cut above lane 1. [file elife-82951-fig5-data1.zip › Figure 5A-Source data/Ben Parker 2022-03-16 15hr 18min_Colourmetric.tif]

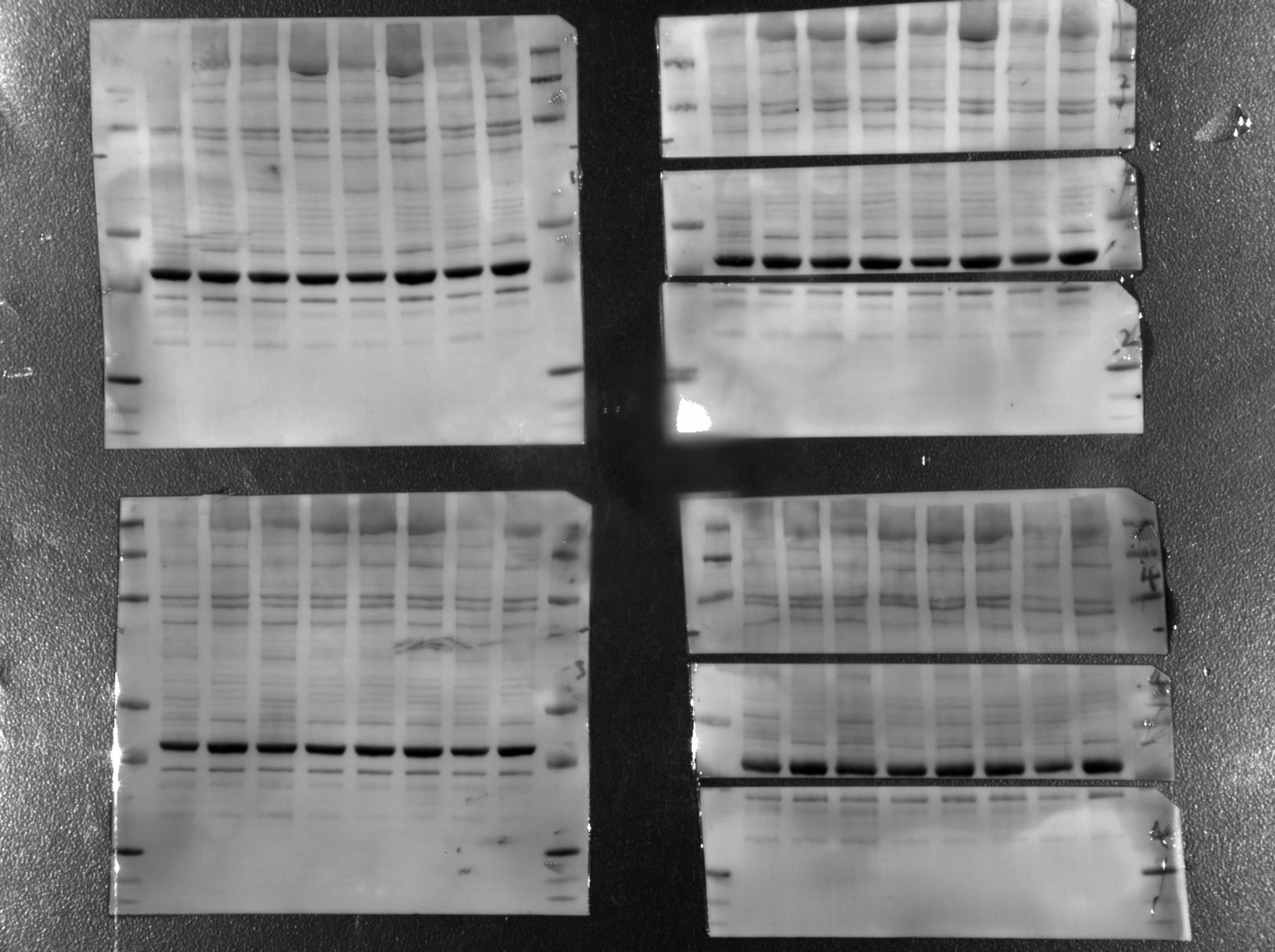

Supplement: Figure 5—source data 1. — The top corner of each membrane is cut above lane 1. [file elife-82951-fig5-data1.zip › Figure 5A-Source data/Yaan-Kit Ng 2022-03-17 13hr 11min_TotalProteinStain.tif]

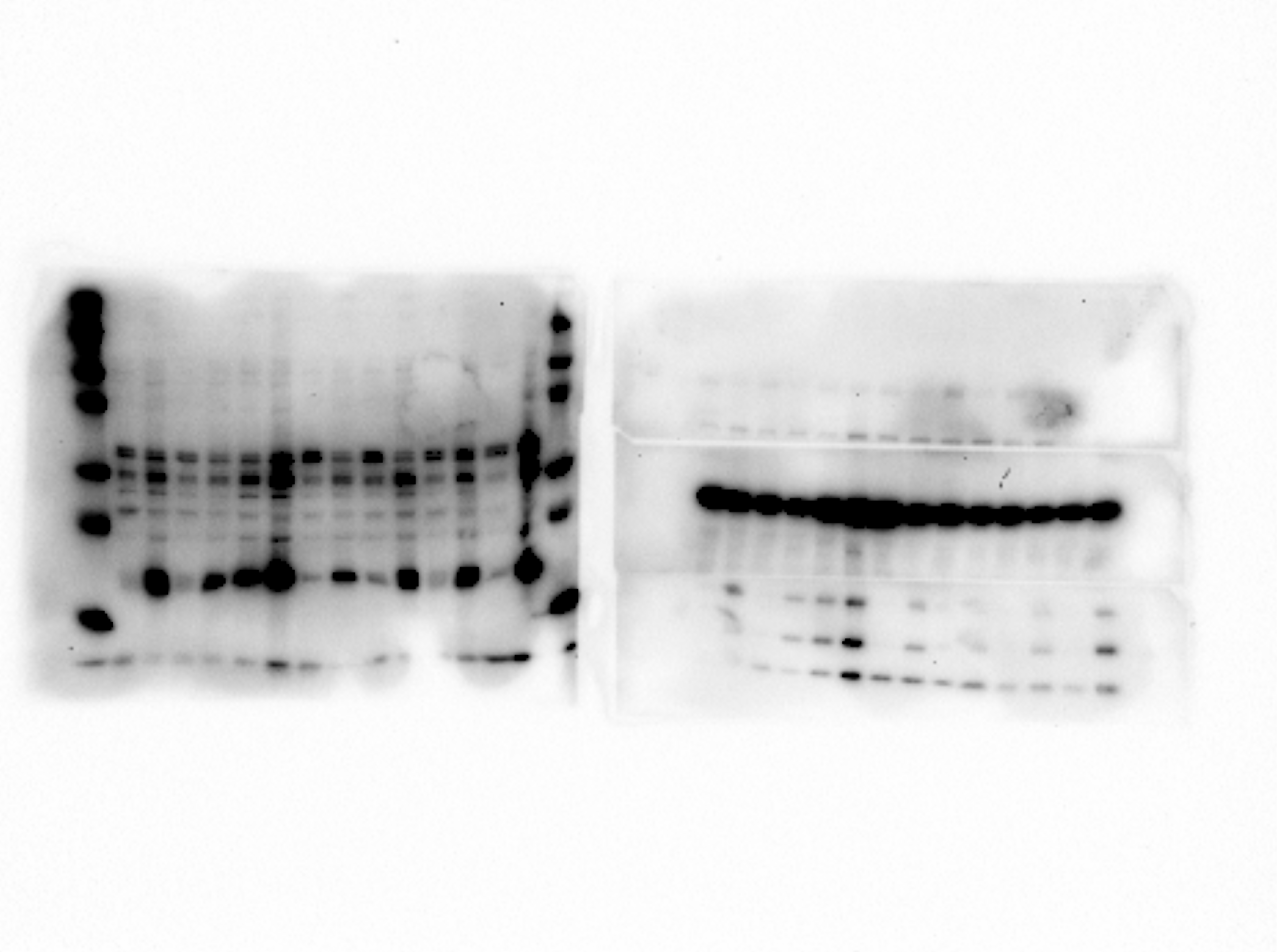

Supplement: Figure 5—source data 2. — The top corner of each membrane is cut above lane 1. [file elife-82951-fig5-data2.zip › Figure 5D-Source data/Ben Parker 2022-06-10 09hr 38min_Exposure_160.6sec.tif]

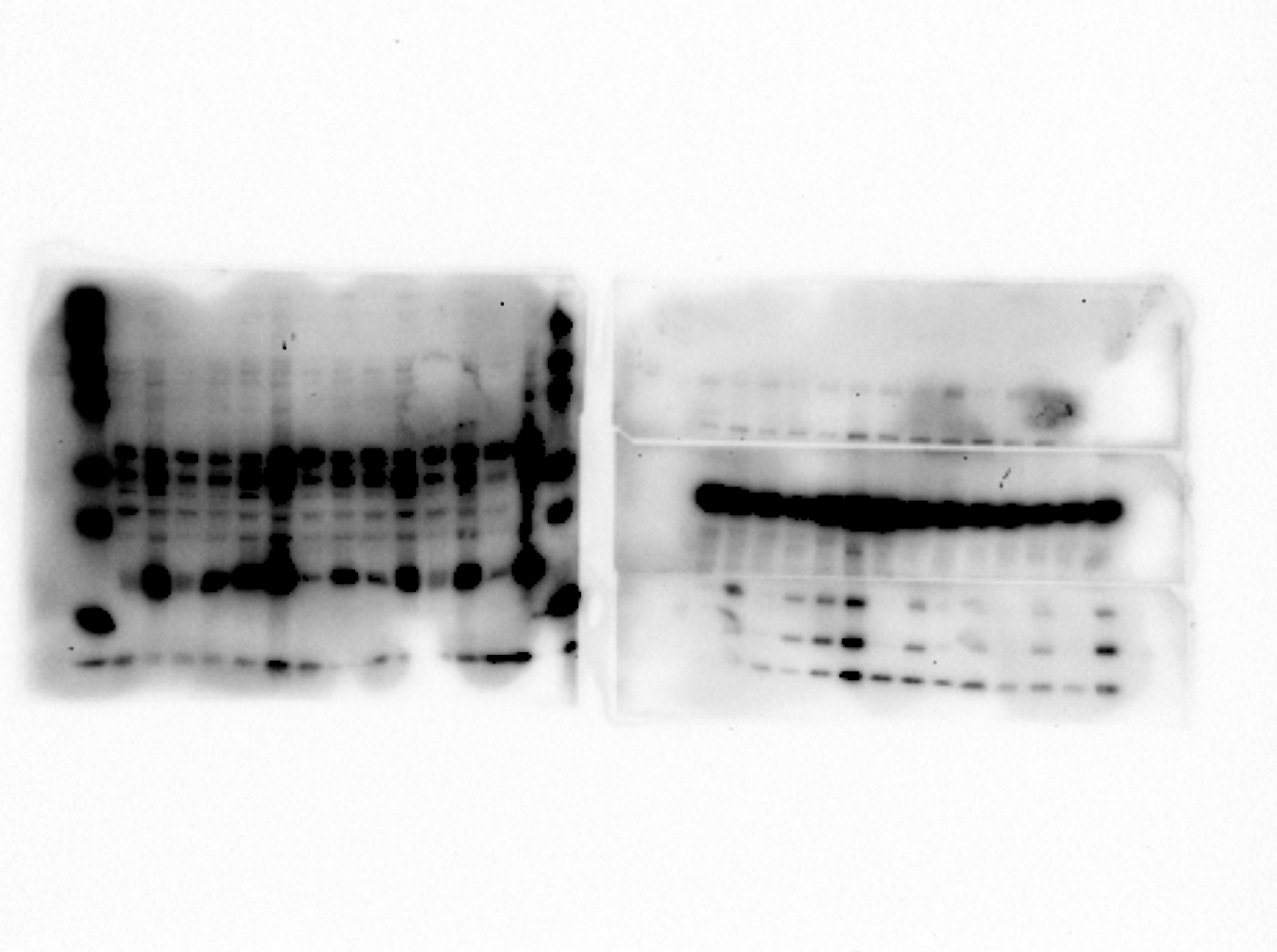

Supplement: Figure 5—source data 2. — The top corner of each membrane is cut above lane 1. [file elife-82951-fig5-data2.zip › Figure 5D-Source data/Ben Parker 2022-06-10 09hr 38min_Exposure_240.3sec.tif]

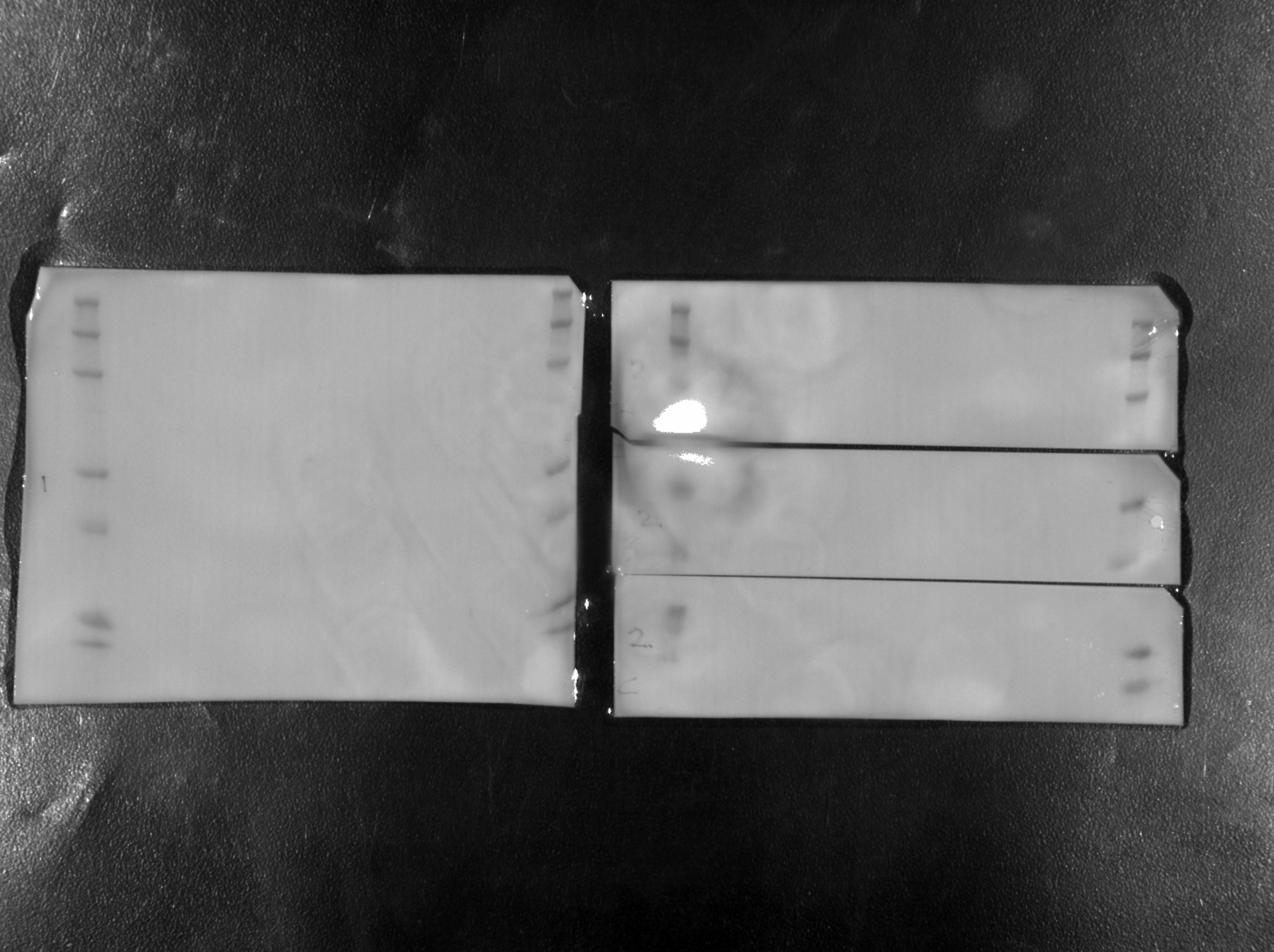

Supplement: Figure 5—source data 2. — The top corner of each membrane is cut above lane 1. [file elife-82951-fig5-data2.zip › Figure 5D-Source data/Ben Parker 2022-06-10 09hr 45min_colourimetric.tif]

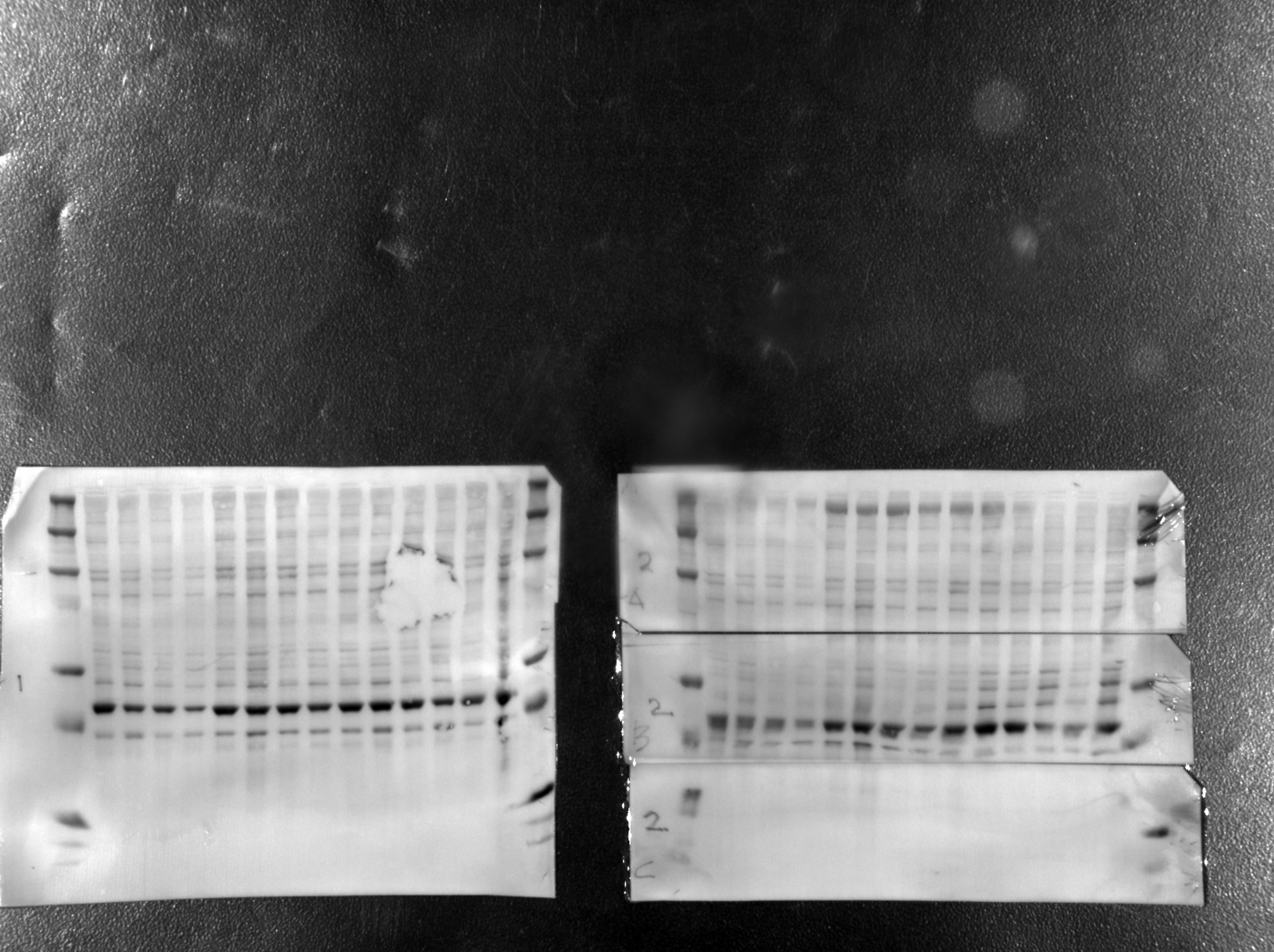

Supplement: Figure 5—source data 2. — The top corner of each membrane is cut above lane 1. [file elife-82951-fig5-data2.zip › Figure 5D-Source data/Ben Parker 2022-06-10 13hr 09min_total protein.tif]

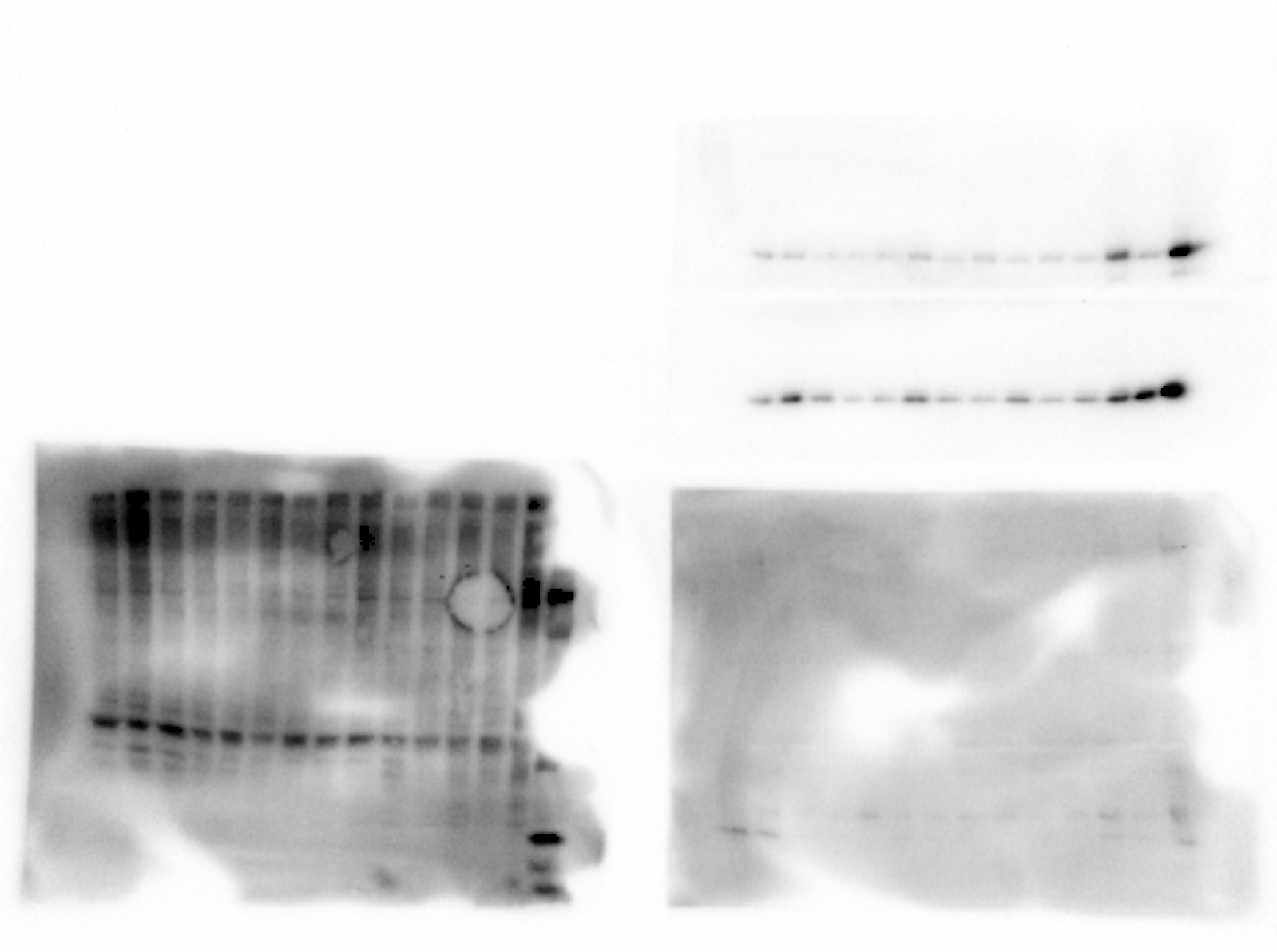

Supplement: Figure 6—source data 1. — The top corner of each membrane is cut above lane 1. [file elife-82951-fig6-data1.zip › Figure 6H-Source data/Ben Parker 2022-07-06 08hr 56min_Exposure_33.8sec.tif]

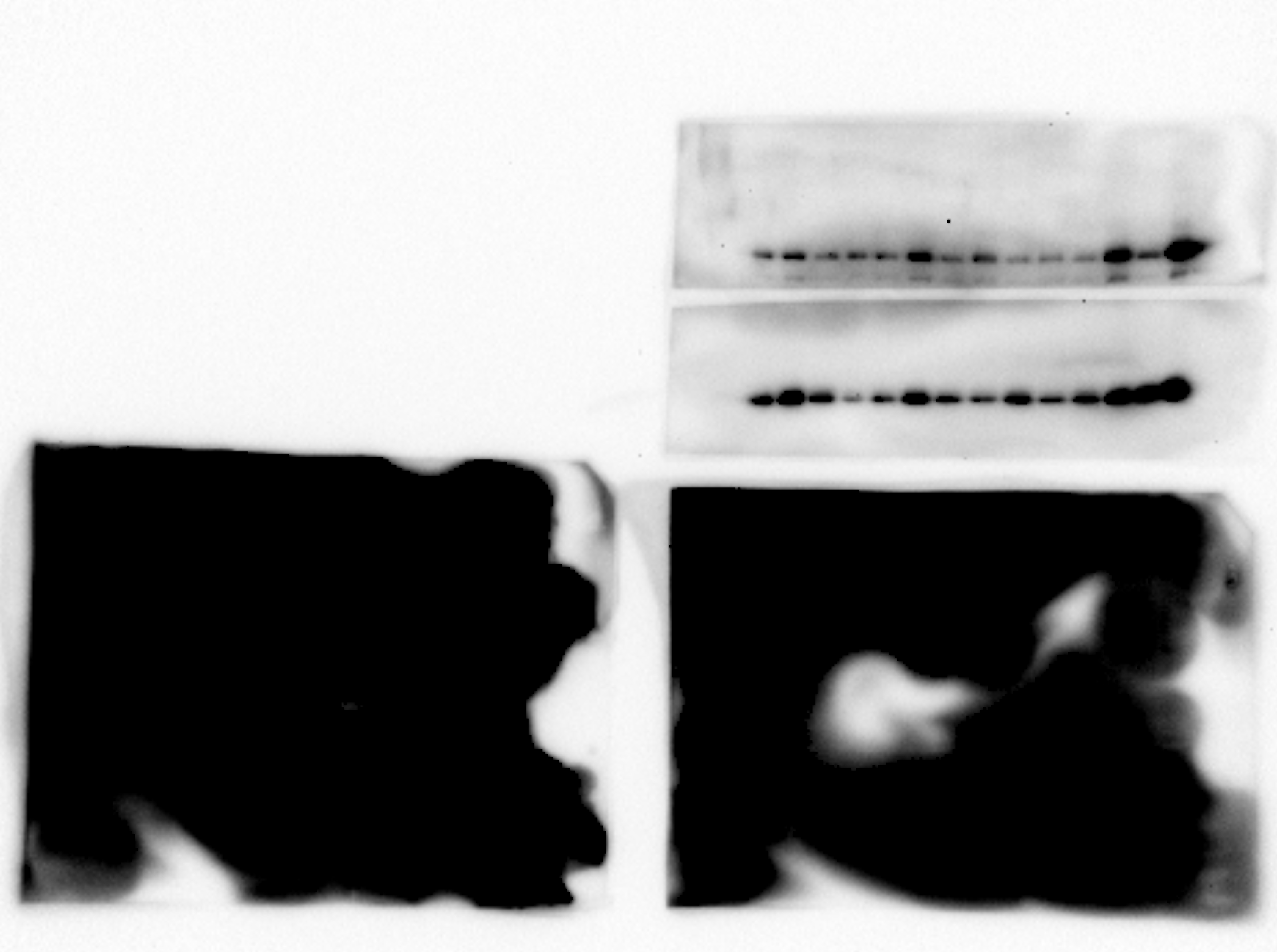

Supplement: Figure 6—source data 1. — The top corner of each membrane is cut above lane 1. [file elife-82951-fig6-data1.zip › Figure 6H-Source data/Ben Parker 2022-07-06 09hr 00min_Exposure_160.3sec.tif]

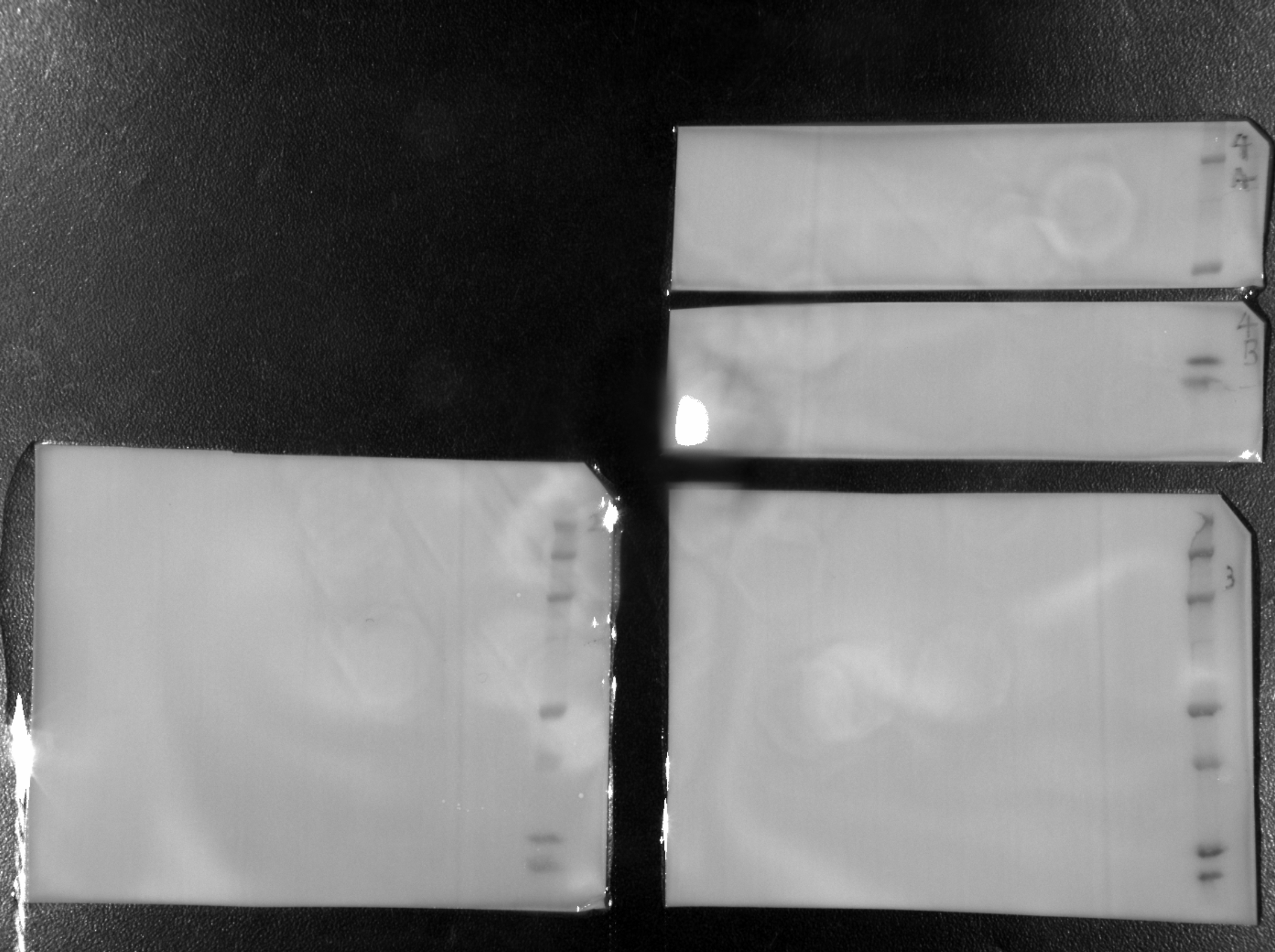

Supplement: Figure 6—source data 1. — The top corner of each membrane is cut above lane 1. [file elife-82951-fig6-data1.zip › Figure 6H-Source data/Ben Parker 2022-07-06 09hr 06min_colourmetric.tif]

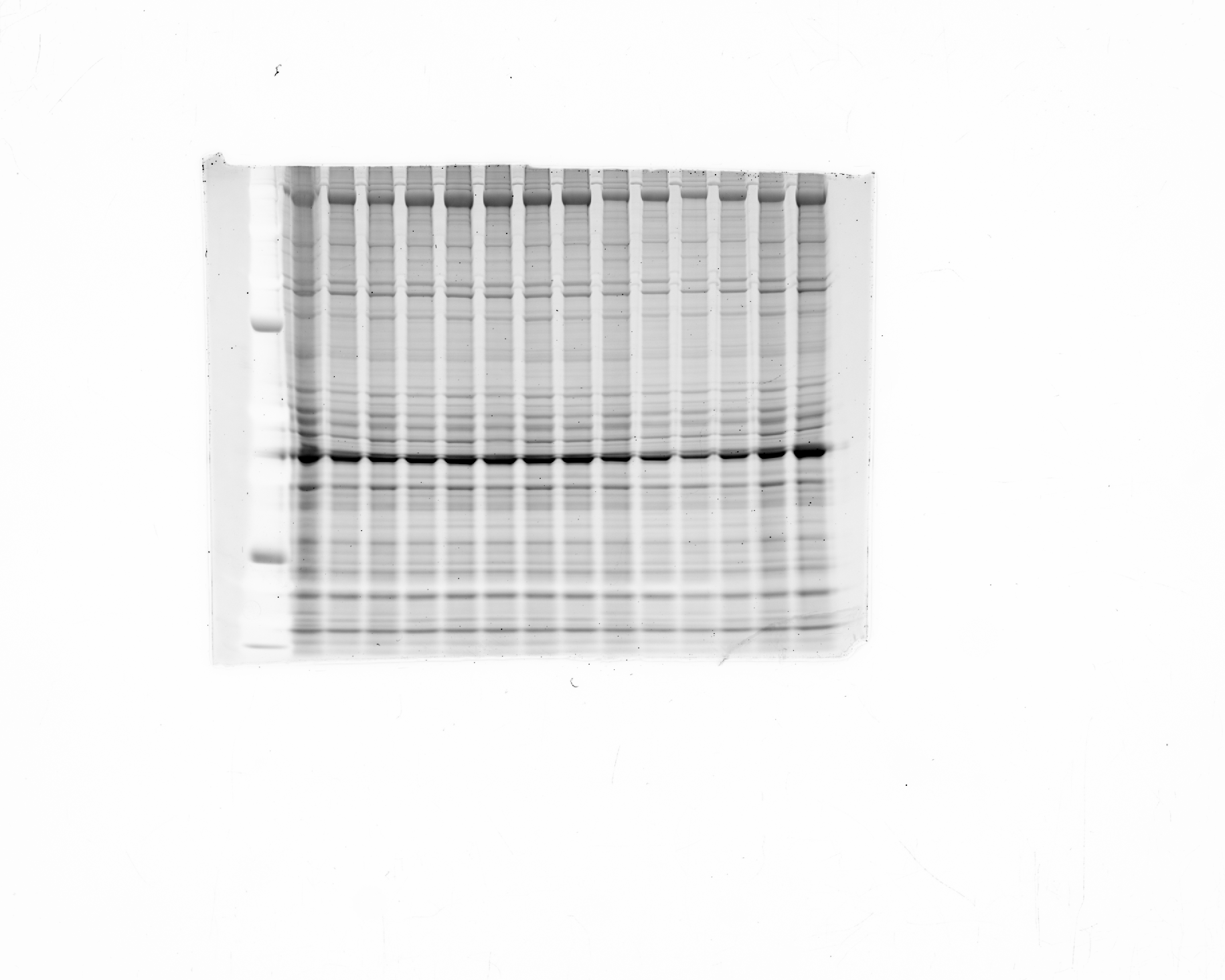

Supplement: Figure 6—source data 1. — The top corner of each membrane is cut above lane 1. [file elife-82951-fig6-data1.zip › Figure 6H-Source data/BenP 2022-07-06 09h32m40s_total protein.tif]
