# Supplementary material for: Proteome-wide systems genetics identifies UFMylation as a regulator of skeletal muscle function
Source: eLife. 2022 Dec 6;11:e82951. doi: 10.7554/eLife.82951 (PMC9833826; doi:10.7554/eLife.82951)
Supplement: Figure 5—source data 2. — The top corner of each membrane is cut above lane 1. [file elife-82951-fig5-data2.zip › Figure 5D-Source data/Figure 5D-source data.pdf]

Figure 5D - Source Data

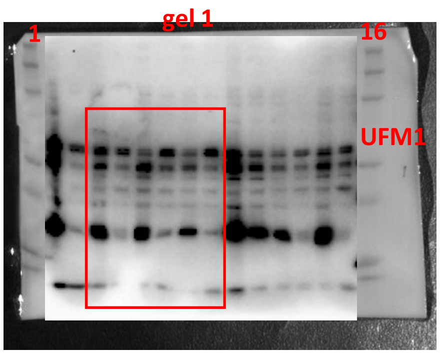

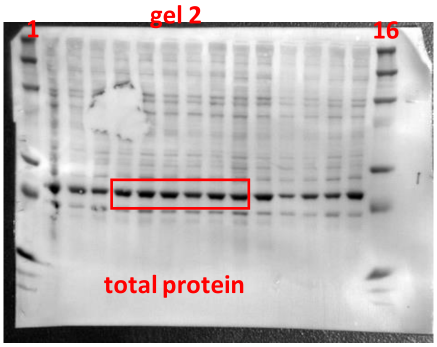

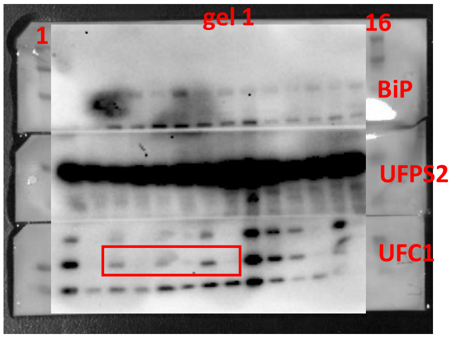

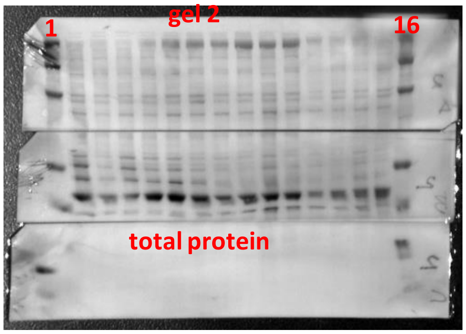

Lane	Mouse ID#_leg	
1	MW marker	
2	435-L	shSramble
3	435-R	shUFC1
4	436-L	shSramble
5	436-R	shUFC1
6	437-L	shSramble
7	437-R	shUFC1
8	438-L	shSramble
9	438-R	shUFC1
10	441-L	shSramble
11	441-R	shUFC1
12	443-L	shSramble
13	443-R	shUFC1
14	444-L	shSramble
15	444-R	shUFC1
16	MW marker	
17		

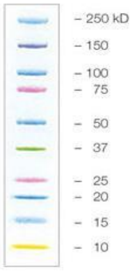

Gel 1

Whole gel

anti-UFM1 (Ab109305; Rb; 1:1000)

Gel 2

cut **under** 75, at 37

anti-BiP (CST3177; 1:1000; RB; 80kDa)

anti-UFSP2 (ab192597; Rabbit; 1:1,000; 53KDa)

anti-UFC1 (Ab189251; Rabbit; 1:10,000; 20kDa)

Mouse ID#_leg	UFC1	total	UFC1/tot prot	UFM1	total	UFM1/total	
435-L	2112772	4754.888	444.3368592	5351.296	4548.153	1.176586628	shSramble
435-R	355760	3852.573	92.34348058	2671.098	3853.518	0.693158304	shUFC1
436-L	735551	4176.024	176.1366793	4202.872	3916.935	1.07300019	shSramble
436-R	361069	4674.844	77.236588	2975.556	3949.21	0.753456008	shUFC1
437-L	789076	5205.634	151.5811523	4252.923	4368.668	0.973505654	shSramble
437-R	368889	5190.196	71.0741945	3261.728	4289.694	0.760363793	shUFC1
438-L	900521	4891.998	184.0804105	3524.994	4024.197	0.875949662	shSramble
438-R	459123	5186.536	88.52208873	3238.465	4309.647	0.751445536	shUFC1
441-L	2747228	5750.645	477.7251943	6046.954	4739.181	1.275949157	shSramble
441-R	1262288	5566.511	226.7646646	4411.411	4469.714	0.986955989	shUFC1
443-L	547030	5030.943	108.7330944	3602.693	3763.938	0.957160559	shSramble
443-R	170020	5256.222	32.34642677	2973.553	3986.156	0.745970052	shUFC1
444-L	1179411	5777.1	204.1527756	4660.083	4368.945	1.066638056	shSramble
444-R	90235	5942.207	15.18543531	3474.886	4697.953	0.739659592	shUFC1
