# Supplementary material for: Proteome-wide systems genetics identifies UFMylation as a regulator of skeletal muscle function
Source: eLife. 2022 Dec 6;11:e82951. doi: 10.7554/eLife.82951 (PMC9833826; doi:10.7554/eLife.82951)
Supplement: Figure 6—source data 1. — The top corner of each membrane is cut above lane 1. [file elife-82951-fig6-data1.zip › Figure 6H-Source data/Figure 6H-source data.pdf]

Figure 6H - Source Data

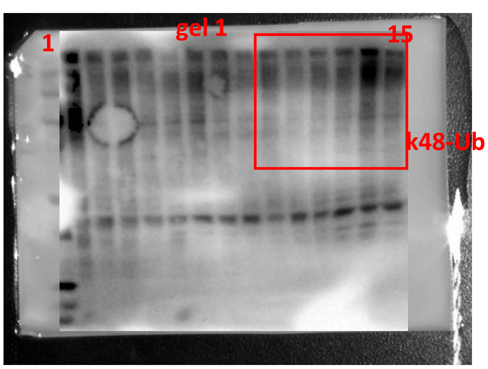

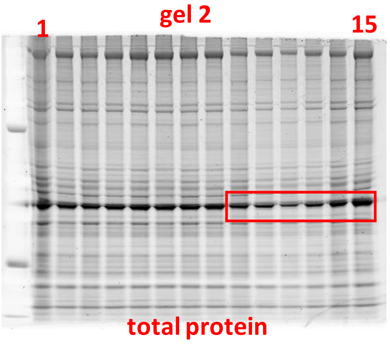

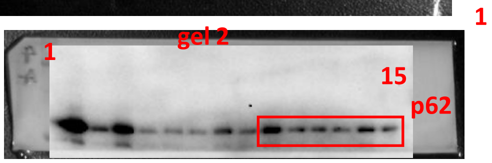

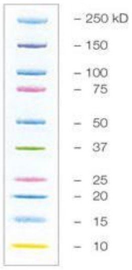

1	MW marker	
2	435-L	shSramble
3	435-R	shUFC1
4	436-L	shSramble
5	436-R	shUFC1
6	437-L	shSramble
7	437-R	shUFC1
8	438-L	shSramble
9	438-R	shUFC1
10	441-L	shSramble
11	441-R	shUFC1
12	443-L	shSramble
13	443-R	shUFC1
14	444-L	shSramble
15	444-R	shUFC1

Gel 1
Whole gel
K48 ubiquitn (CST:8081; 1:1000; RB)

gel 2
cut 50kDa
SQSTM1/p62 (CST:8025; 1:1000; RB; 62 kDa)

1	MW marker		total	p62	p62/total	K48	K48/total
2	435-L	shSramble	4101.392	6521718	1.590123061	4163114	1.015049037
3	435-R	shUFC1	2826.636	2362460	0.835785011	2664168	0.94252249
4	436-L	shSramble	3067.099	4012953	1.308387176	3204595	1.044829332
5	436-R	shUFC1	2752.215	1055322	0.383444607	1655372	0.601468999
6	437-L	shSramble	3022.341	963920	0.318931583	1252060	0.414268277
7	437-R	shUFC1	2960.981	800377	0.27030805	2678556	0.90461776
8	438-L	shSramble	3001.246	1376130	0.458519562	1952216	0.650468505
9	438-R	shUFC1	2705.43	1188276	0.439218904	917618	0.339176397
10	441-L	shSramble	2667.067	2033413	0.762415417	2340094	0.87740353
11	441-R	shUFC1	1965.605	952910	0.484792214	1400443	0.712474276
12	443-L	shSramble	1826.305	1012013	0.554131429	1920451	1.051549988
13	443-R	shUFC1	2154.666	1071772	0.49741909	1645377	0.763634364
14	444-L	shSramble	3040.557	1266717	0.416606891	3765214	1.238330345
15	444-R	shUFC1	3512.913	1078769	0.30708674	1513082	0.430720032
